# Successful treatment for adrenocorticotropic hormone-independent macronodular adrenal hyperplasia with laparoscopic adrenalectomy: a case series

**DOI:** 10.1186/1752-1947-6-312

**Published:** 2012-09-18

**Authors:** Toshiki Ito, Yutaka Kurita, Hitoshi Shinbo, Atsushi Otsuka, Hiroshi Furuse, Soichi Mugiya, Tomomi Ushiyama, Seiichiro Ozono, Yutaka Oki, Kazuo Suzuki

**Affiliations:** 1 Department of Urology, JA Shizuoka Kohseiren Enshu Hospital, 1-1-1 Chuo, Naka-ku, Hamamatsu, Shizuoka, 430-0929, Japan; 2 Department of Urology, Hamamatsu University School of Medicine, 1-20-1 Handayama, Higashi-ku, Hamamatsu, Shizuoka, 431-3192, Japan; 3 Department of Endocrinology & Metabolism, Hamamatsu University School of Medicine, 1-20-1 Handayama, Higashi-ku, Hamamatsu, Shizuoka, 431-3192, Japan; 4 Department of Urology, Institute of Minimally Invasive Surgery, Shintoshi Hospital, 703 Nakaizumi, Iwata, Shizuoka, 438-0078, Japan

## Abstract

**Introduction:**

Adrenocorticotropic hormone-independent macronodular adrenal hyperplasia, characterized by bilateral macronodular adrenal hypertrophy and autonomous cortisol production, is a rare cause of Cushing’s syndrome. Bilateral adrenalectomy is considered the standard treatment for adrenocorticotropic hormone-independent macronodular adrenal hyperplasia but obliges the patient to receive lifetime steroid replacement therapy subsequently, and may increase the patient’s risk of adrenal insufficiency. These circumstances require surgeons to carefully consider operative strategies on an individual basis.

**Case presentation:**

We performed successful laparoscopic adrenalectomy on four patients with adrenocorticotropic hormone-independent macronodular adrenal hyperplasia. Computed tomography scans showed bilateral adrenal enlargement in all patients. Case 1: a 56-year-old Japanese woman presented with obvious Cushing’s symptoms during treatment for diabetes mellitus and hypertension. Case 2: a 37-year-old Japanese man also presented with Cushing’s symptoms during treatment for diabetes mellitus and hypertension. These patients were diagnosed as Cushing’s syndrome caused by adrenocorticotropic hormone-independent macronodular adrenal hyperplasia based on endocrinologic testing, and underwent bilateral laparoscopic adrenalectomy. Case 3: an 80-year-old Japanese woman was hospitalized due to unusual weight gain and heightened general fatigue, and was diagnosed as Cushing’s syndrome caused by adrenocorticotropic hormone-independent macronodular adrenal hyperplasia. She underwent unilateral laparoscopic adrenalectomy due to high operative risk. Case 4: a 66-year-old Japanese man was discovered to have bilateral adrenal tumors on medical examination. He did not have Cushing’s symptoms and was diagnosed as subclinical Cushing’s syndrome due to suppressed adrenocorticotropic hormone serum levels and loss of cortisol circadian rhythm without abnormal levels of serum cortisol. He underwent unilateral laparoscopic adrenalectomy. During follow-up, serum cortisol levels were within the normal range in all cases, and serum adrenocorticotropic hormone levels were not suppressed. Further, cases with Cushing’s syndrome experienced clinical improvement.

**Conclusions:**

We were able to effectively treat adrenocorticotropic hormone-independent macronodular adrenal hyperplasia in patients with obvious Cushing’s symptoms by laparoscopic bilateral adrenalectomy, which promptly improved symptoms. Further, unilateral adrenalectomy was effective for treating an older patient at high operative risk and a patient with subclinical Cushing’s syndrome.

## Introduction

Adrenocorticotropic hormone (ACTH)-independent macronodular adrenal hyperplasia (AIMAH), an impairment demonstrated by bilateral macronodular adrenal hypertrophy and autonomous cortisol production, is a rare cause of Cushing’s syndrome. Although bilateral adrenalectomy is considered the standard treatment for AIMAH [1,2], patients receiving this treatment are subsequently obliged to receive lifetime steroid replacement therapy and may be susceptible to adrenal insufficiency. Given these concerns, the operative strategy for treating AIMAH must be considered carefully for each individual case.

Here we report the surgical and clinical aspects of four cases of AIMAH that were treated by laparoscopic adrenalectomy, including two requiring bilateral adrenalectomy.

## Case presentations

**Figure 1:**
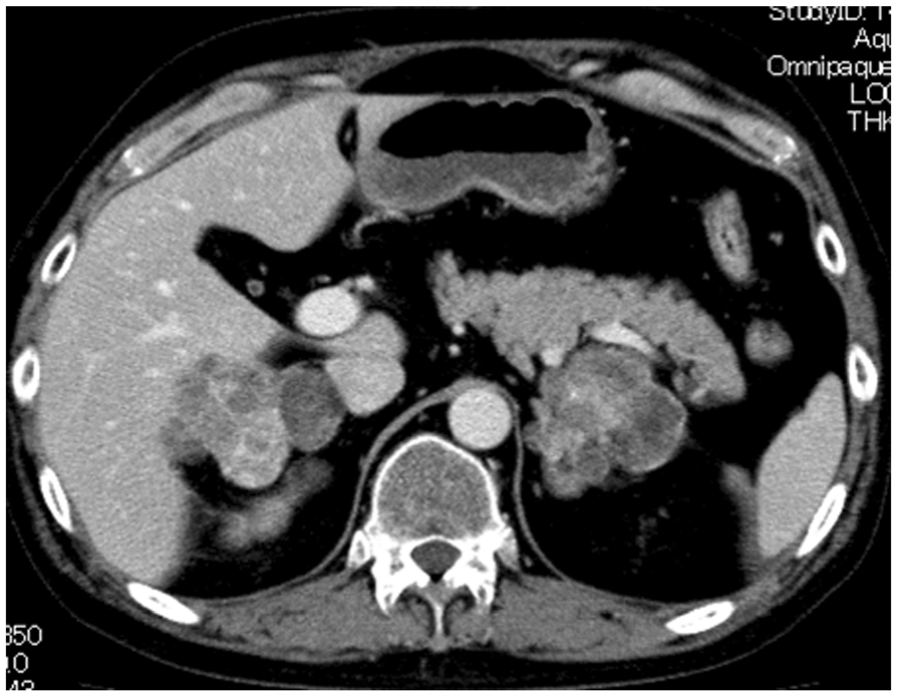
Abdominal computed tomography in patient 2 showing bilateral multinodular adrenal enlargement with maximum diameters of 8.5cm and 10.5cm on the right and left sides, respectively.

We performed adrenalectomies on four consecutive patients with AIMAH from 1999 to 2010. The patients’ clinical and biochemical findings are summarized in Table 1. Enhanced computed tomography (CT) showed bilateral adrenal enlargement with slight enhancement (Figure 1), and adrenal scintigraphy revealed bilateral uptake of ^131^I-norcholesterol was observed in all patients.

**Table 1:** Clinical and biochemical findings

**Case**	**Age**	**Gender**	**Body mass index (kg/m**^**2**^**)**	**Size of adrenal gland** **(L/R, cm)**	**Cushing’s symptoms**	**Serum cortisol (μg/dL) (basal values: 5.3 to 11.0)**		**Plasma adrenocorticotropic hormone (pg/mL) (normal values: 7.2 to 63.3)**
						**At 8 a.m.**	**At 11 p.m.**	
1	56	F	21.2	6.5/5.5	+	21.5	20.4	<5.0
2	37	M	26.6	10.5/8.5	+	34.1	28.7	<5.0
3	80	F	21.7	4.0/3.5	+	14.2	16.2	<5.0
4	66	M	29.6	6.0/8.5	-	10.4	10.7	<5.0

### Case 1

A 56-year-old Japanese woman presented with several symptoms characteristic of Cushing’s syndrome, including moon face and central obesity, during treatment for diabetes mellitus and hypertension. She was diagnosed as Cushing’s syndrome caused by AIMAH due to autonomous production of adrenal cortisol, which was accompanied by suppressed serum ACTH levels (<5.0pg/mL) and a loss of cortisol circadian rhythm. She underwent laparoscopic bilateral adrenalectomy because of her overt Cushing’s symptoms. The operative procedure in this case has been described by Shinbo *et al*. [3]. Cortisol replacement therapy was started immediately upon completion of surgery.

### Case 2

**Figure 2:**
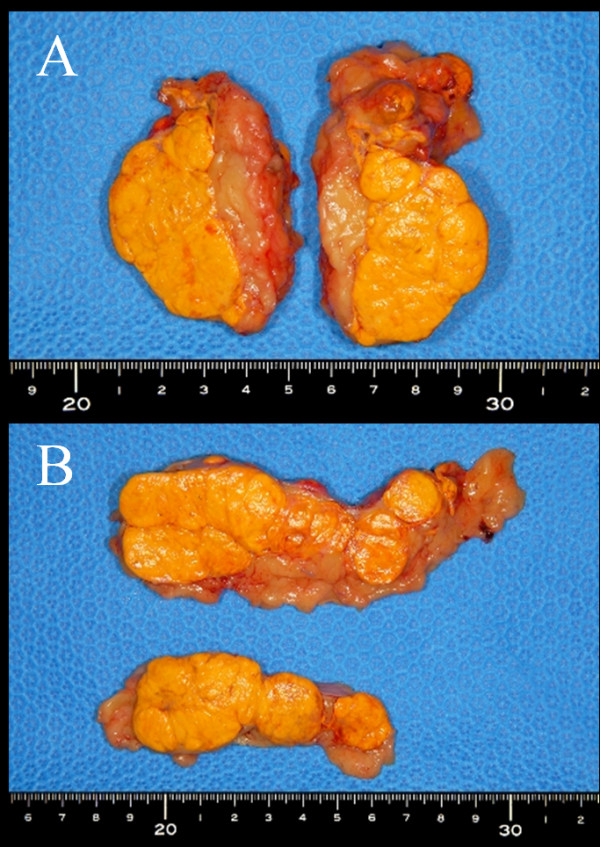
**Macroscopic appearance of the adrenal glands of patient 2.** (**A**) The right gland weighed 141g and measured 85×60×40mm. (**B**) The left gland weighed 145g and measured 105×45×40mm. Both glands exhibited many macronodules on the exposed surface.

A 37-year-old Japanese man presented with severe Cushing’s symptoms during treatment for diabetes mellitus and hypertension. Symptoms included moon face, central obesity, and muscle weakness. He underwent laparoscopic bilateral adrenalectomy and cortisol replacement therapy post-operatively (Figure 2).

### Case 3

An 80-year-old Japanese woman was hospitalized because of unusual weight gain (about 10kg over about two years) and general fatigue. Upon admission, she was diagnosed as Cushing’s syndrome. She underwent unilateral adrenalectomy due to high operative risk. We based our decision on the side to operate on based on which gland was larger according to CT scan findings.

### Case 4

A 66-year-old Japanese man was incidentally discovered to have bilateral adrenal tumors but no Cushing’s symptoms. The endocrinological data showed a normal level of cortisol (10.4μg/dL) with a suppressed serum ACTH level (<5.0pg/mL), which showed neither a cortisol circadian rhythm nor suppression by dexamethasone administration (9.1μg/dL after 1mg; 16.9μg/dL after 8mg). He underwent right laparoscopic adrenalectomy due to subclinical Cushing’s syndrome. In this case, the right adrenal gland was removed, because adrenal venous sampling revealed that cortisol production from the right gland was much greater than from the left (right: 38.9μg/dL; left: 20.3μg/dL).

The surgical procedures and results are summarized in Table 2. The duration of the operations for patients 1 and 2 included the time required to change the patient’s position. The estimated blood loss was for patient 1 was 350mL and negligible in the others. All patients were able to walk and receive oral nutrient intake by post-operative day 2. Hydrocortisone was administered intravenously to patients 1 and 2 starting on the day of the operation. The drug was administered orally after the patients stared taking meals orally. The dose was tapered to 15 or 20mg/day until discharge, which prolonged the length of their hospitalization. No conversions to open surgery were performed, and no severe complications occurred during the post-operative period.

**Table 2:** Operative procedures and results

**Case**	**Site of adrenalectomy**	**Operation time (minutes)**	**Blood loss (g)**	**Weight of adrenal gland (L/R, g)**	**Discharge (post-operative day)**	**Dosage of hydrocortisone at discharge (mg/day)**	**Complications**
1	Bilateral	424	350	57/51	20	20	Surgical site infection
2	Bilateral	520	Negligible	145/141	18	15	None
3	Left	134	Negligible	30/-	7	None	None
4	Right	244	Negligible	-/50	6	None	None

Clinical courses and outcomes are summarized in Table 3. Clinical improvements, such as disappearance of moon face, central obesity, and muscle weakness were evident in patients 1, 2, and 3. Patient 4 remained asymptomatic for Cushing’s syndrome during follow-up. Serum cortisol levels have remained within normal ranges and serum ACTH levels were unsuppressed in all cases during follow-up.

**Table 3:** Clinical course and outcomes

**Case**	**Follow-up duration (months)**	**Dosage of hydrocortisone (mg/day)**	**Cushing's symptoms**	**Body mass index (kg/m**^**2**^**)**	**Endocrinological examination**	
					**Serum cortisol at 8 a.m. (μg/dL) (normal values: 5.3 to 11.0)**	**Plasma adrenocorticotropic hormone (pg/mL) (normal values: 7.2 to 63.3)**
1	146	15	Improved	20.5	2.7	25.7
2	35	15	Improved	24.2	1.1	50.1
3	24	None	Improved	20.3	11.2	7.9
4	90	None	Subclinical	28.3	14.6	4.1

## Discussion

Bilateral adrenalectomy and steroid replacement therapy is considered the standard treatment for AIMAH [1,2]. Several cases of successful laparoscopic bilateral adrenalectomy for AIMAH have been reported [3-6], and each involved little blood loss during surgery. Laparoscopic adrenalectomy is less invasive than open surgery for treating AIMAH and may prevent a number of post-operative complications due to impaired glucose tolerance and immunodeficiency. However, treatment of AIMAH requires considerable skill given the impressive size and fragility of adrenal tumors.

Although we opted for a bilateral approach here, several patients have been treated successfully after receiving only medical treatment or subtotal or unilateral adrenalectomy [7-9]. The rationale for implementing these treatments was to avoid impairing our patients’ quality of life due to the risk of critical adrenal insufficiency after bilateral adrenalectomy. Two recent reports note that unilateral adrenalectomy of the larger gland with AIMAH resulted in improvement in Cushing’s symptoms after a mean follow-up time of 53 or 78.8 months [10,11]. However, the efficacy of unilateral adrenalectomy for AIMAH remains controversial because the mean patient age in these reports is 52.6 years, clearly insufficient for long-term assessment. We reasoned, therefore, that if a patient receives a second operation to remove the remaining gland when Cushing’s syndrome recurs during follow-up, unilateral adrenalectomy might also be effective in selected cases such as those reported here. In patients with subclinical AIMAH, the decision regarding therapy should take into account normalization of cortisol excess. Unilateral adrenalectomy is the treatment of choice, as well as medical treatment or subtotal or unilateral adrenalectomy, for normalization of cortisol excess. However, careful attention must be paid after unilateral adrenalectomy, as the criteria and long-term prognosis of this procedure in subclinical AIMAH have not been well established. If unilateral adrenalectomy is performed, determination of the surgical site should be based on asymmetrical adrenal enlargement or adrenal venous sampling analysis; the gland more strongly influencing the patient’s condition should be removed.

## Conclusions

In our experience, laparoscopic bilateral adrenalectomy for AIMAH with obvious Cushing’s symptoms was effective in rapidly improving symptoms when performed by an experienced surgical team. Further, unilateral adrenalectomy can also be effective when the patient is older and subject to high operative risk or does not present with clinical symptoms. Because long-term prognosis associated with this surgical technique remains controversial, strategies for treating AIMAH must be carefully considered on a case-by-case basis.

## Consent

Written informed consent was obtained from all patients for publication of this case report and accompanying images. A copy of the written consent is available for review by the Editor-in-Chief of this journal.

## Competing interests

The authors declare that they have no competing interests.

## Authors’ contributions

TI and YK wrote the manuscript. TI, YK, AO, HS, HF, SM, TU, and KS cared for our patients. YO performed endocrinological examinations and patient management. All authors reviewed the report and approved the final version of the manuscript.
